# Supplementary material for: Impact of prognostic nutritional index on outcomes in patients with Mycobacterium avium complex pulmonary disease
Source: PLoS One. 2020 May 6;15(5):e0232714. doi: 10.1371/journal.pone.0232714 (PMC7202629; doi:10.1371/journal.pone.0232714)
Supplement: S1 Table — (DOCX) [file pone.0232714.s001.docx]

**Supporting information**

**S1 Table.**

| Variables | OR (95% CI) | P-value |
| --- | --- | --- |
| Age ≥ 65 years | 1.142 (0.592-2.203) | 0.693 |
| Gender, Male | 1.371 (0.696-2.699) | 0.362 |
| Cavitary lesion on computed tomography | 1.456 (0.771-2.751) | 0.247 |
| AFB Smear |  |  |
| Negative | Reference |  |
| 1+ or 2+ | 2.838 (1.153-6.985) | 0.023 |
| 3+ or 4+ | 1.418 (0.386-5.207) | 0.599 |
| Time from diagnosis to treatment, months | 0.982 (0.962-1.003) | 0.091 |
| Malnutrition (PNI* <45) | 1.288 (0.630-2.637) | 0.488 |

*List of Abbreviations*: OR, odds ratio; 95% CI, 95% confidence interval; AFB, acid-fast bacilli; PNI, prognostic nutritional index

*PNI = 10 × serum albumin value (g/dL) + 0.005 × total lymphocyte count in the peripheral blood (/mm^3^)
