# Supplementary material for: Impact of prognostic nutritional index on outcomes in patients with Mycobacterium avium complex pulmonary disease
Source: PLoS One. 2020 May 6;15(5):e0232714. doi: 10.1371/journal.pone.0232714 (PMC7202629; doi:10.1371/journal.pone.0232714)
Supplement: Attachment — Submitted filename: Response to the reviewers.docx [file pone.0232714.s003.docx]

**Response to the Reviewers**

**Title: Impact of prognostic nutritional index on outcomes in patients with Mycobacterium avium complex pulmonary disease**

**Journal: Plos One
Submission ID:** **PONE-D-19-31183**

Sung Woo Moon, Eun Hye Lee, Ji Soo Choi, Ah Young Leem, Su Hwan Lee, Sang Hoon Lee, Song Yee Kim, Kyung Soo Chung, Ji Ye Jung, Moo Suk Park, Young Sam Kim, Young Ae Kang

**Reviewer #1**

1. Authors reported useufullness of PNI in MAC-PD patients. Low PNI patients failed treatment and showed poor prognosis. Cut-off value of PNI should be calculated by ROC cureve or other methods. However, cut-off value of PNI was determined by other reports.

**Reply:** Thank you for your kind advice. We have used receiver operating characteristic (ROC) curve for mortality to calculate the cut-off of PNI.

The cut-off was 46.2 (Sensitivity 57.14, Specificity 77.53, AUC 0.698, P < 0.001). When using this cut-off, multivariate logistic regression analyses for variables related to treatment intolerance (Table 3), multivariate logistic regression analyses for variables related to culture conversion failure (S1 Table), and multivariate Cox’s proportional hazard analyses of factors for mortality in *Mycobacterium avium complex* lung disease patients (part of Table 5) changed as follows.

**Table 3. Multivariate logistic regression analyses for variables related to treatment intolerance**

| Variables | OR (95% CI) | P-value |
| --- | --- | --- |
| Age ≥ 65 years | 1.367 (0.950-1.969) | 0.092 |
| Gender, Male | 0.847 (0.586-1.225) | 0.377 |
| Dyspnea | 0.907 (0.515-1.598) | 0.735 |
| Hemoptysis | 0.547 (0.353-0.847) | 0.547 |
| Malnutrition, treatment initiation (PNI <46.2) | 1.866 (1.186-2.937) | 0.007 |

**S1 Table. Multivariate logistic regression analyses for variables related to culture conversion failure**

| Variables | OR (95% CI) | P-value |
| --- | --- | --- |
| Age ≥ 65 years | 1.135 (0.587-2.194) | 0.707 |
| Gender, Male | 1.246 (0.628-2.474) | 0.529 |
| Cavitary lesion on computed tomography | 1.415 (.0746-2.685) | 0.288 |
| Positive AFB Smear | 2.350 (1.069-5.163) | 0.033 |
| Time from diagnosis to treatment, months | 0.981 (0.961-1.002) | 0.081 |
| Malnutrition (PNI* <45) | 1.236 (0.595-2.567) | 0.571 |

**Table 5. Univariate and multivariate Cox’s proportional hazard analyses of factors for mortality in Mycobacterium avium complex lung disease patients**

| Variables | Multivariate | | |
| --- | --- | --- | --- |
|  | HR (95% CI) | P-value |  |
| Age ≥ 65 years | 1.908 (0.998-3.649) | 0.051 |  |
| Sex, male | 2.379 (1.289-4.393) | 0.006 |  |
| Body mass index, kg/m^2^ | 0.866 (0.793-0.946) | 0.001 |  |
| Positive AFB smear | 2.838 (1.576-5.111) | 0.001 |  |
| Malnutrition, Diagnosis (PNI < 46.2) | 2.812 (1.623-4.872) | <0.001 |  |
| Treatment started | 0.559 (0.321-0.973) | 0.040 |  |
| Hypertension | 1.208 (0.659-2.215) | 0.542 |  |
| Diabetes | 1.356 (0.726-2.567) | 0.350 |  |
| Chronic kidney disease | 1.709 (0.682-4.277) | 0.253 |  |
| Cardiovascular disease | 1.218 (0.630-2.355) | 0.558 |  |
| Cancer | 2.443 (1.410-4.233) | 0.001 |  |

As demonstrated in the above results, low PNI is a risk factor for mortality and treatment intolerance. PNI did not change and the consistent reliability of PNI in MAC-PD was confirmed. But the problem with using ROC curve in defining cut-off is that through the paper, we wanted to analyze not only mortality but also intolerance and treatment response, and we thought that using multiple cut-off could be confusing. Also, the cut-off of 45 was used as the cut-off 45 was not verified in MAC-PD patients. However, we believe that a prospective study on MAC-PD patients, that will clarify a new cut-off for malnutrition is needed. The sentence in discussion section has been changed. (Page 16, line 1, 2)

**Reviewer #2**

This study is a retrospective cohort analysis to evaluate whether patients with malnutrition, as defined by PNI, are high odds of intolerance to treatment, lower odds of achieving culture conversion after treatment, and higher risk of mortality than non-malnutrition patients.

I think this study is very interesting because this is the first study to analysis the relationship between PNI and outcome of MAC-PD.

I would like to request some revisions to make the article more sophisticated.

1. The authors concluded that malnutrition as defined by PNI is an independent predictor for treatment intolerance and all-cause mortality in patients with MAC-PD.

In table 1, there are many differences in the background between non-malnutrition group and malnutrition group. Although the authors performed a multivariate analysis, first of all, can malnutrition be an independent factor because there are so many compounding factors.

As you know, it is natural that patients with malnutrition are high age, easy to discontinue the treatment of MAC-PD, and therefore, they have poor prognosis.

The impact of the primary outcome in this study may be slightly weak.

**Reply:** We totally agree with your valuable advice. Due to the retrospective nature of the study and the limitation of the number of MAC-PD patients, we could not match variables nor add more variables between the groups. Thus, we used univariate Cox regression analysis first and then put the meaningful variables in the multivariate analysis. Thus, we explored whether malnutrition can be an independent factor excluding many compounding factors. Additionally, we also checked the multicollinearity between the variables. We used the term ‘related’ rather than ‘associated’ and the term ‘associated’ was changed to ‘related’ and added the point made by the reviewer as part of the limitation of the study. (Page 15, line 21 and Page 17, line 13-15)

2. As the disease of MAC-PD progresses, the nutrition status will get worsens.

I doubt that the patients with malnutrition group had more severe MAC-PD compared to non-malnutrition group. I think that the description regarding the status of the MAC-PD at diagnosis is poor.

**Reply:** Thank you for your advice. Until now, there has been no established way to evaluate MAC-PD's severity, but currently, the presence of cavitary lesion on chest CT and sputum AFB smears are frequently used to examine the severity of MAC-PD in clinical practice. In our analysis, we included the presence of cavitary lesion on chest CT. Additionally, AFB smear was also included in the analysis, but AFB smear was only categorized into positive or negative. To give additional data on the status of MAC-PD at diagnosis, the AFB status was additionally categorized into “negative”, “1+ and 2+”, and “3+ and 4+”. We consider this could give additional data on the severity of MAC-PD. Analyses were performed again and the numbers in the paper have changed accordingly. (Abstract, Result section, Tables, S1 Table, Page 4, line 21-23)

**Reviewer #3**

This study evaluated the impact of PNI on outcomes in patients with MAC PD.

The manuscript was well written.

I have only a few comments.

Major

1. In Introduction section, please add the reason why you chose to evaluate PNI among the various nutrition assessment tools in this study.

**Reply:** We have added the reason why we chose to evaluate PNI among the various nutrition assessment tools in the introduction and the discussion sections. (Page 3, line 7-8)

Minor

1. page 12 line 11 "Based off" →　You mean "Based on" ？

**Reply:** Thank you for your correction. We have changed “Based off” to “Based on”. (Page 6, line 21)

Again, we appreciate the opportunity to revise out manuscript, and we hope our revision meets your approval. Sincerely yours,

Sincerely,

Young Ae Kang, M.D., PhD.

Division of Pulmonary Medicine, Department of Internal Medicine

Yonsei University College of Medicine, 50 Yonsei-ro, Seodaemun-gu, Seoul 120-752, Republic of Korea

Tel: +82-2-2228-1940

Fax: +82-2-393-6884

E-mail: mdkang@yuhs.ac
